# Mind the Difference Between Primary Tics and Functional Tic‐like Behaviors

**DOI:** 10.1002/mds.28853

**Published:** 2021-12-17

**Authors:** Kirsten R. Müller‐Vahl, Mark J. Edwards

**Affiliations:** ^1^ Clinic of Psychiatry, Social Psychiatry and Psychotherapy Hannover Medical School Hannover Germany; ^2^ Institute of Molecular and Clinical Sciences St George's University of London London United Kingdom

Beginning in spring 2019, movement disorder specialists around the world who specialize in diagnosis and treatment of people with tic disorders have seen the emergence of a new phenomenon of acute‐onset tic‐like behaviors in young people. Although initially it has been speculated that symptoms may represent “tic attacks” in patients with tic disorders or a rare variant of Tourette syndrome with acute onset,^1^, ^2^ soon it became clear that these acute‐onset tic‐like symptoms are functional in nature.^3^ The emergence of this new and so far unknown presentation of a functional neurological disorder (FND) has been linked, on the one hand, to social media use and presentation of tic‐like behavior of popular YouTube and TikTok influencers^3^, ^4^ and, on the other hand, to consequences of the coronavirus disease 2019 (COVID‐19) pandemic.^5^ Over the past 2 years, the world has been through an incredible period of change. Normal activities and behaviors such as going to school or work, travel, meeting others face‐to‐face, and even hugging our loved ones have been stopped or significantly curtailed. These restrictions have occurred in a climate of fear and uncertainty: fear and uncertainty for personal and family health, economic survival, and the future of society as we (used to) know it. The impacts of death, illness, and sudden restriction of freedoms continue to reverberate throughout local, national, and international society.

Two articles^6^, ^7^ in this issue of *Movement Disorders* address this important and emerging phenomenon of acute‐onset tic‐like behaviors and seek to understand its causes and treatment.

Pringsheim et al^6^ describe a cohort of 290 young people in the Calgary Tic Disorders Registry gathered between 2012 and June 2021. They compared 20 patients from the registry who had rapid onset of tic‐like behaviors from March 2020 onward (ie, during the pandemic) with the remainder of the cohort. Those with tic‐like behaviors were older, more likely to be female, and had higher severity as rated by the Yale Global Tic Severity Scale. Tic‐like movements and vocalizations tended to be more complex with a lack of typical rostrocaudal distribution of movements, and most had copro‐phenomena with a large number of bizarre phrases, swear words, insults and offensive statements, and self‐injurious or externally injurious movements. When controlling for age and sex, there was a significant relationship between a diagnosis of functional tic‐like behaviors and anxiety and depressive disorders. All those with such behaviors reported accessing content on social media (mainly TikTok) relating to people claiming to suffer from tics or Tourette syndrome.

Paulus et al^7^ describe a cohort of 13 patients with rapid‐onset tic‐like behaviors that the authors also link to exposure to social media, particularly the YouTube Channel “Gewitter im Kopf” (“Thunderstorm in the head”), where a young man presents with symptoms atypical for Tourette syndrome, such as complex, variable, often continuous movements and elaborated and variable swear words and offensive phrases. Demographic and clinical features were similar to the Pringsheim et al^6^ cohort with respect to age at onset, severity of symptoms, and predominance of complex motor and vocal phenomena predominantly involving the trunk and arms and lack of waxing and waning. Different from Pringsheim et al's study,^6^ the group comprised more males than females, although the ratio was smaller than seen in Tourette samples. In this small group of patients, several clinical features were not different between this sample and those with Tourette syndrome, including premonitory sensations, ability to suppress movements and vocalizations, as well as history (self‐reported) of autistic spectrum disorder, attention deficit/hyperactivity disorder, and obsessive‐compulsive disorder. The authors did not formally assess levels of anxiety and depression.

What do these two cohorts tell us when added together with previously published work on sudden onset of tic‐like behaviors^1^, ^3^, ^8^ and previous work on functional tic‐like symptoms more generally^9^?

First, they clearly document an explosive presentation of a FND with “Tourette‐like” symptoms. Although at first glance, some similarities with clinical presentation of Tourette syndrome can be found, a closer look, however, reveals several obvious differences, including age of onset, course of symptoms, and number and kind of movement and vocalization. Because most of these patients had been misdiagnosed with Tourette syndrome before being seen at the specialist center, it is of utmost importance not to *automatically* make the diagnosis of Tourette syndrome in all those patients with otherwise unexplained jerks, vocalizations, and copro‐phenomena, but also to consider the diagnosis of a functional disorder. The typical onset of tics is discrete with simple motor tics such as eye blinking between the age of 5 and 7 years with typical waxing and waning course thereafter. In other words, rapid onset of tic‐like symptoms with complex movements and swear words well after the age of 10 years with constant symptom progression excludes the diagnosis of Tourette syndrome.

Second, although patients with functional tic‐like behaviors were generally experiencing symptoms of a higher severity than patients with Tourette syndrome, in many cases an incongruity between reported severity and function is obvious. For example, patients and their families often report that because of the symptoms, unwanted obligations can no longer be performed, while favorite activities can be conducted without any restrictions. Some of the patients even started to present their own symptoms on social media.

Third, there is an interesting association between development of functional tic‐like behaviors and depressive symptoms and anxiety, suggesting that these symptoms may be an additional risk factor for the development of functional “Tourette‐like” behaviors.

Fourth, early diagnosis is key, and these publications will help in the recognition of the phenotype and early diagnostic explanation. Accelerating the diagnostic and diagnostic explanation process is likely to lead to better outcomes going by previous evidence in people with FND.^10^ Some clinicians have reported that symptoms may even stop immediately after exclusion of the diagnosis of Tourette syndrome and adequate explanation and education.

Fifth, there is increasing evidence that people with Tourette syndrome may also develop rapid‐onset functional tic‐like behaviors. Such an overlap between Tourette syndrome and FND has been reported before in a small number of cases. It is possible that in the past this co‐incidence of tics and functional tic‐like symptoms has been overlooked in a substantial proportion of patients with Tourette syndrome, potentially leading to escalation of medical (and even consideration of surgical) treatment.

Lastly, there is the issue of social drivers to illness and the expression of illness. This is not a new phenomenon, as seen in mass sociogenic illnesses, some of which have involved tic‐like behaviors.^11^, ^12^ Recently, the term *mass social media–induced illness* has been suggested for this new type of mass sociogenic illness spread solely via social media.^3^ Indeed, authors have noted the presence of rapid‐onset tic‐like behaviors in the context of social media viewing before the pandemic.^3^, ^4^ The pervasive, international nature of social media massively increases the potential reach of (mis)information and the generation of illness expectations and beliefs that are incorrect and that may trigger and escalate symptoms, particularly in those who are vulnerable. This process also harms a major medical benefit of the information revolution provided by the internet and social media. This benefit is the easy access to reliable information about medical problems and the dissemination of self‐management and other therapeutic advice. As highlighted in the article by Paulus et al,^7^ Tourette patient organizations have pushed back against the depictions on social media of people with functional tic‐like behaviors as being typical of Tourette syndrome, and other organizations and societies, such as the International Parkinson's and Movement Disorder Society, could possibly do the same. Movement disorders in those with functional movement disorders are often severe, and the uncertainty and lack of support that often characterizes the relationship between such patients and healthcare professionals may lead them to be more likely to post videos of themselves online, seeking help and validation.^13^ Previous work has highlighted this on YouTube, where the most popular videos related to different movement disorders were judged by experts to be most likely videos of people with functional movement disorders. Interestingly, in this study from 2011, the only movement disorder category where this was not the case was people with tics.^13^ Perhaps, at least in part, the patients reported in these two reports represent a move toward the norm of functional movement disorders on social media.

## Author Roles

(1) Manuscript: A. Writing of the first draft, B. Review and Critique.

K.R.M.‐V.: 1A, 1B

M.J.E.: 1A, 1B

## Financial Disclosures

KMV has received financial or material research support from EU (FP7‐HEALTH‐2011 No. 278367, FP7‐PEOPLE‐2012‐ITN No. 316978), DFG: GZ MU 1527/3‐1 and GZ MU 1527/3‐2, BMBF: 01KG1421, National Institute of Mental Health (NIMH), Tourette Gesellschaft Deutschland e.V., Else‐Kröner‐Fresenius‐Stiftung, GW pharmaceuticals, Almirall Hermal GmbH, Abide Therapeutics, and Therapix Biosiences. She has received consultant's honoraria from Abide Therapeutics, Boehringer Ingelheim International GmbH, Bionorica Ethics GmbH, CannaMedical Pharma GmbH, Canopy Grouth, Columbia Care, CTC Communications Corp., Demecan, Ethypharm GmbH, Eurox Deutschland GmbH, Global Praxis Group Limited, Lundbeck, MCI Germany, Neuraxpharm, Sanity Group, Stadapharm GmbH, Synendos Therapeutics AG, and Tilray. She has received royalties from Deutsches Ärzteblatt, Der Neurologie und Psychiater, Elsevier, Medizinisch Wissenschaftliche Verlagsgesellschaft Berlin, and Kohlhammer.

ME has received funding from NIHR. He has received honoraria for educational events from Merz Pharma and payments from Willey for Editorial work for European Journal of Neurology.

## Data Availability

Data sharing not applicable to this article as no datasets were generated or analysed during the current study.
